# Relationship between anti-thyroid peroxidase antibody positivity and pregnancy-related and fetal outcomes in Euthyroid women: a single-center cohort study

**DOI:** 10.1186/s12884-020-03176-4

**Published:** 2020-08-26

**Authors:** Ning Yuan, Jianbin Sun, Zhi Li, Sanbao Chai, Xiaomei Zhang, Linong Ji

**Affiliations:** 1 Department of Endocrinology, Peking University People’s Hospital, Beijing, 100044 China; 2 Department of Endocrinology, Peking University International Hospital, Beijing, 102206 China; 3 Department of gynaecology and obstetrics, Peking University International Hospital, Beijing, 102206 China

**Keywords:** Thyroid peroxidase antibodies, Euthyroid, Pregnancy and fetal outcomes

## Abstract

**Background:**

Thyroid autoimmunity (TAI) and subclinical hypothyroidism (SCH) have been associated with poor pregnancy and fetal outcomes. However, whether euthyroid women with anti-thyroid peroxidase antibody (TPOAb) positivity have a higher risk of poor pregnancy and fetal outcomes is debatable. Therefore, this study aimed to investigate the association between TPOAb positivity and pregnancy-related and fetal outcomes in euthyroid women.

**Methods:**

In total, 938 pregnant women participated in this prospective cohort study. The euthyroid group included 837 pregnant women and the TPOAb-positive group included 101 euthyroid pregnant women. Serum TPOAb, thyroglobulin antibody (TGAb), thyroid-stimulating hormone (TSH), and free thyroxine (FT4) levels were assessed. Pregnancy and fetal outcomes included gestational diabetes mellitus, spontaneous abortion, premature rupture of membranes, hypertensive disorders of pregnancy, preterm birth, fetal distress, low birth weight, fetal macrosomia, and small for gestational age infant.

**Results:**

Logistic regression analysis showed TPOAb positivity was not associated with an increased risk of poor pregnancy or fetal outcomes in euthyroid women. However, TPOAb-positive euthyroid women pregnant with a female fetus were independently associated with preterm births (OR: 4.511, 95% CI: 1.075–18.926) after adjustment for potential confounding factors.

**Conclusions:**

TPOAb positivity was not found to be associated with poor pregnancy-related or fetal outcomes in euthyroid women. However, in euthyroid women with a female fetus, TPOAb positivity was strongly associated with preterm births. The risk of preterm birth in the euthyroid women with TPOAb positivity should be emphasized in clinical practice.

**Trial registration:**

ClinicalTrials.gov Identifier: NCT02966405. Registered on October 24th 2016 - Retrospectively registered.

## Background

Recent studies have reported that thyroid autoimmunity (TAI) and subclinical hypothyroidism (SCH) are associated with an increased risk of poor pregnancy and fetal outcomes, such as spontaneous abortion, preterm birth, and lower offspring motor and intelligence quotients [1, 2]. TAI is characterized by anti-thyroid peroxidase antibody (TPOAb) and/or thyroglobulin antibody (TGAb) positivity. Guidelines published by the American Thyroid Association (ATA) in 2017 [3] recommended establishing population-based trimester-specific reference ranges for serum thyroid-stimulating hormone (TSH) levels. If information is unavailable, the recommended upper normal limit cutoff for TSH is 4.0 mU/L. Meanwhile, 2.5 mU/L can be used as the cutoff for SCH combined with TPOAb positivity. Levothyroxine (LT4) replacement therapy may be considered for TPOAb-positive women with TSH concentrations > 2.5 mU/L and below the upper limit of the pregnancy-specific reference range [3]. However, whether TPOAb positivity is associated with poor pregnancy and fetal outcomes in euthyroid women is debatable. It is known that the prevalence of TPOAb varies with ethnicity, gender, and iodine intake [3–5]. A recent meta-analysis [6] showed the presence of TAI was associated with an increased risk of spontaneous miscarriage. A separate meta-analysis [7] found TPOAb-positive women had a higher risk of preterm birth than TPOAb-negative women. Several studies have examined the associations between maternal TAI and child autism spectrum [8] and lower motor and intellectual development [9]. TPOAb positivity has been associated with poor pregnancy and fetal outcomes in euthyroid women in some but not all studies [10, 11]. Therefore, the aim of the present study was to investigate the impact of TPOAb positivity on the pregnancy-related and fetal outcomes in euthyroid women.

## Methods

### Study population

**Fig. 1:**
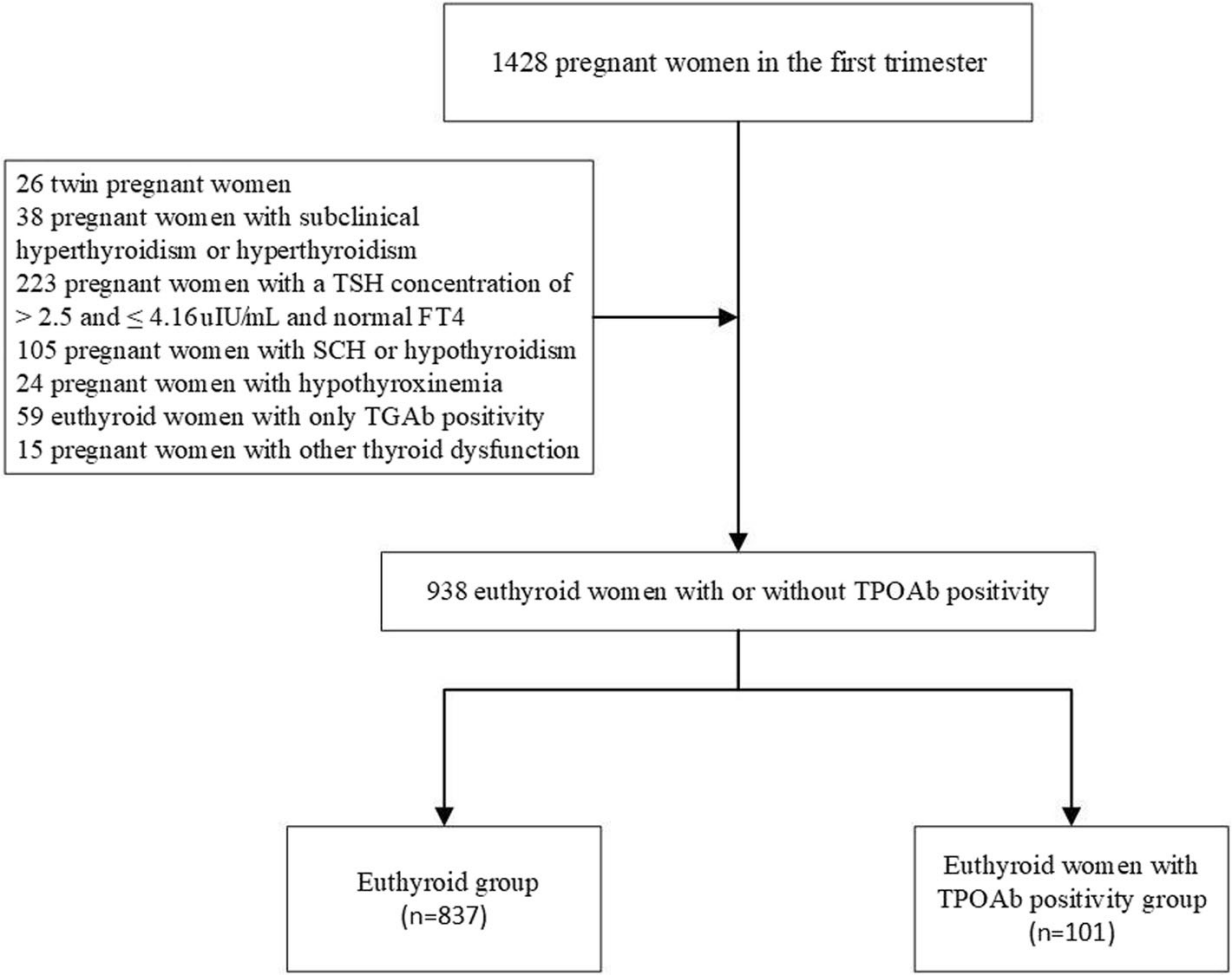
Flow chart of patient selection. TSH, thyroid-stimulating hormone; FT4, free thyroxine; SCH, subclinical hypothyroidism; TGAb, thyroglobulin antibody; TPOAb, thyroid peroxidase antibody

This was a prospective cohort study conducted at a single site. The inclusion criteria were (a) single birth; (b) fetus at 4–8 weeks’ gestation diagnosed by last menstrual period and human chorionic gonadotropin (HCG); (c) euthyroid women (TSH > 0.12 and ≤ 2.5 uIU/mL) in the first trimester of pregnancy. The exclusion criteria were (a) diagnosis of hereditary disease, tumors, autoimmune disease (e.g., systemic lupus erythematosus, Sjogren syndrome, or antiphospholipid antibody syndrome), heart disease, liver disease, renal disease, or chronic hypertension; (b) consumption of medication that could affect thyroid function. In total, 1428 pregnant women who presented at the Department of Gynaecology and Obstetrics of Peking University International Hospital volunteered to participate in this study from October 2016 to April 2018. In total, twenty six women with twin pregnancies, 38 pregnant women with subclinical hyperthyroidism or hyperthyroidism, 223 pregnant women with a TSH concentration of > 2.5 and ≤ 4.16 uIU/mL, and normal FT4, 105 pregnant women with SCH or hypothyroidism, 24 pregnant women with hypothyroxinemia, 59 euthyroid women with only TGAb positivity, and 15 pregnant women with other thyroid dysfunctions were excluded. Ultimately, 938 subjects participated in this study. The euthyroid group included 837 pregnant women with a TSH concentration of > 0.12 and ≤ 2.5 uIU/mL, normal FT4, and TPOAb and TGAb negativity. The TPOAb-positive group included 101 TPOAb-positive with or without TGAb positivity euthyroid women (Fig. 1). Written informed consents were obtained from all study participants. This study was registered on ClinicalTrials.gov (Identifier: NCT02966405).

All pregnant women completed the questionnaire about the history of thyroid or autoimmune disease, family health history of thyroid disorders, and previous abortion. The height, weight, blood pressure, and gestational age of the participants were recorded. The body-mass index (BMI) was calculated as weight in kilograms divided by height in meters squared. The serum blood glucose (GS), glycosylated hemoglobin (HbA1c), low-density lipoprotein cholesterol (LDL-C), uric acid (UA), homocysteine (Hcy) and ferritin tests were analyzed and recorded. The participants were followed up through the second and third trimesters of pregnancy until delivery. The pregnancy status of participants was recorded, including the mode of birth, delivery time, neonatal gender, weight and height, and other pregnancy-related data. The pregnancy-related and fetal outcomes included gestational diabetes mellitus (GDM), spontaneous abortion, premature rupture of membranes (PROM), hypertensive disorders of pregnancy (HDP), preterm birth, fetal distress, low birth weight, fetal macrosomia, and small for gestational age (SGA) infant.

### Definitions

GDM was diagnosed if one or more of the following plasma GS levels were met or exceeded following the 75 g oral glucose tolerance test: at fasting ≥92 mg/dL, at 1 h ≥ 180 mg/dL, and at 2 h ≥ 153 mg/dL, according to the guidelines of the International Association of Diabetes and Pregnancy Study Groups [12]. Spontaneous abortion was defined as a spontaneous loss of a pregnancy before the 20th week of gestation [13]. PROM was defined as the rupture of the amniotic sac before the onset of labor. HDP included gestational hypertension, preeclampsia-eclampsia, chronic hypertension (of any cause diagnosed before 20 weeks of gestation), and chronic hypertension with preeclampsia superimposed [14, 15]. Preterm birth was defined as the birth of a baby at fewer than 37 weeks’ gestation excluding iatrogenic preterm birth caused by preeclampsia, placenta previa, fetal growth restriction, and other factors. Fetal distress was defined as a fetus suffering from insufficient oxygen supply, based on abnormal fetal heart rate and movements, or acidosis (fetal scalp blood sample showing pH < 7.20) [16]. Low birth weight was defined as a live birth smaller than 2500 g. Fetal macrosomia was defined as a live birth of more than 4000 g. SGA infant was defined by a birth weight below the 10th percentile of normative birth weights for singletons [17].

### Thyroid function tests

TSH, FT4, TGAb, and TPOAb from collected serum samples were assessed using a COBAS Elesys 601 immunoassay analyzer (Roche Diagnostics, Switzerland). The intraassay coefficient of variability (CV) for serum TSH, FT4 and TPOAb were 1.1–3.0%, 1.5–4.3% and 2.7–6.3% respectively, and the interassay CVs were 3.2–7.2%, 3.3–8.4% and 4.2–9.5%, respectively. According to pregnancy-specific thyroid function guidelines established in Peking University International Hospital, the reference range of thyroid indicators for the 2.5th to 97.5th percentile for TSH level was 0.12 uIU/mL to 4.16 uIU/mL and for the FT4 level, it was 13.36 pmol/L to 23.55 pmol/L. A TPOAb titer of 34 IU/mL or more and a TGAb titer of 115 IU/mL or more were classified as positivity (in non-pregnant women).

### Outcome parameters

The primary aim of the present study was to investigate the impact of TPOAb positivity on preterm birth in euthyroid women. The secondary objectives were to explore the role of TPOAb positivity on other pregnancy-related and fetal outcomes, and to compare the effect of TPOAb positivity on pregnancy-related and fetal outcomes according to fetal gender.

### Sample size calculation

According to previous studies [7], it was assumed for the sample size calculation that the rate of preterm births in the TPOAb-positive group were 6.6 and 4.9% in the euthyroid group resulting in a minimum sample size of 803 pregnant women for a two-sided chi-square test with a power of 0.80 and a two-sided t-test with a significance level of 5%. The rate of non-response in this study was estimated to be 10%, therefore, requiring a sample size at least 892 cases.

### Statistical analysis

We used SPSS version 17.0 (SPSS, IBM, USA) for all statistical analyses. Continuous data conforming to the normal distribution were expressed as number ± standard deviation; otherwise, they were expressed as median (interquartile range [IQR]). Categorical data were expressed as numbers (percentages) of cases. Differences between the euthyroid group and the TPOAb-positive euthyroid group were analyzed by a t-test or Mann-Whitney U test for continuous data and by a Fisher’s exact test for categorical data. Univariable logistic regression analysis was used to assess the association between demographic parameters and pregnancy and fetal outcomes. A multivariate logistic regression model was used to assess the association between TPOAb-positive euthyroid women and pregnancy and fetal outcomes. In the model, the confounding factors for adjustment included TSH and HbA1c, as well as demographic parameters such as age, BMI, parity, and history of spontaneous abortion. A *P*-value < 0.05 was considered statistically significant.

## Results

### Characteristics of participants

The characteristics of the pregnant women in this study are listed in Table 1. There were 837 pregnant women in the euthyroid group and 101 pregnant women in the TPOAb-positive euthyroid group. The serum TSH levels were higher in the TPOAb-positive euthyroid group than in the euthyroid group (median TSH 1.51 uIU/ml, IQR [1.14–2.08] uIU/ml vs. 1.31 uIU/ml, IQR [0.826–1.80] uIU/ml, *P* < 0.001). No significant differences were observed between the two groups regarding maternal age, BMI, the rate of parity and history of spontaneous abortion, or the serum levels of HbA1c, GS, LDL, UA, Hcy, ferritin, and FT4.

**Table 1:** Subject’s characteristics in Euthyroid group and Euthyroid women with TPOAb positivity group

	Euthyroid group (*n* = 837)	Euthyroid women with TPOAb positivity group (*n* = 101)	Statistics	P
Maternal age (years)	30 (28,34)	31 (29,34)	−1.660	0.097
Age ≥ 35 years (%)	157 (18.8%)	21 (20.8%)	0.243	0.622
BMI (kg/m^2^)	21.48 (19.81,23.43)	21.35 (20.07,23.19)	−0.149	0.881
BMI ≥ 24 kg/m^2^ (%)	174 (20.8%)	19 (18.8%)	0.215	0.643
Parity (%)				
primipara	519 (62.0%)	60 (59.4%)	0.258	0.611
multipara	318 (38.0%)	41 (40.6%)		
History of spontaneous abortion (%)	114 (13.6%)	11 (10.9%)	0.581	0.446
HbA1c (%)	5.1 (4.9,5.3)	5.1 (5.0,5.3)	−1.174	0.240
GS (mmol/L)	4.88 (4.65,5.14)	4.85 (4.65,5.15)	−0.175	0.861
TSH (uIU/ml)	1.31 (0.826,1.80)	1.51 (1.14,2.08)	−3.793	0.000*
FT4 (pmol/L)	17.20 (15.80,18.50)	16.9 (15.80,18.80)	−0.318	0.751
LDL (mmol/L)	2.02 (1.69,2.37)	1.98 (1.77,2.28)	−0.426	0.670
UA (μmol/L)	209 (181,236)	215 (189,239)	−0.841	0.401
Hcy (μmol/L)	6.40 (5.70,7.20)	6.1 (5.70,7.38)	−0.224	0.823
Ferritin (ng/ml)	53.1 (33.03,80.95)	48.75 (29.95,69.30)	−1.426	0.154

*P < 0.05

**Statistics:** Maternal age, BMI, FT4, TSH, GS, HbA1c, LDL, UA, Hcy and Ferritin for Mann-Whitney U test; Age<35 years, BMI<24 kg/m^2^, parity, and history of spontaneous abortion for chi-square test or Fisher test

Continuous data are expressed as median (interquartile range). Categorical data are expressed as numbers (percentages) of cases

*BMI* body-mass index, *TSH* thyroid-stimulating hormone, *FT4* free thyroxine, *HbA1c* glycated hemoglobin, *GS* blood glucose, *LDL* low density lipoprotein cholesterol, *UA* uric acid, *Hcy* homocysteine

### Pregnancy and fetal outcomes

**Fig. 2:**
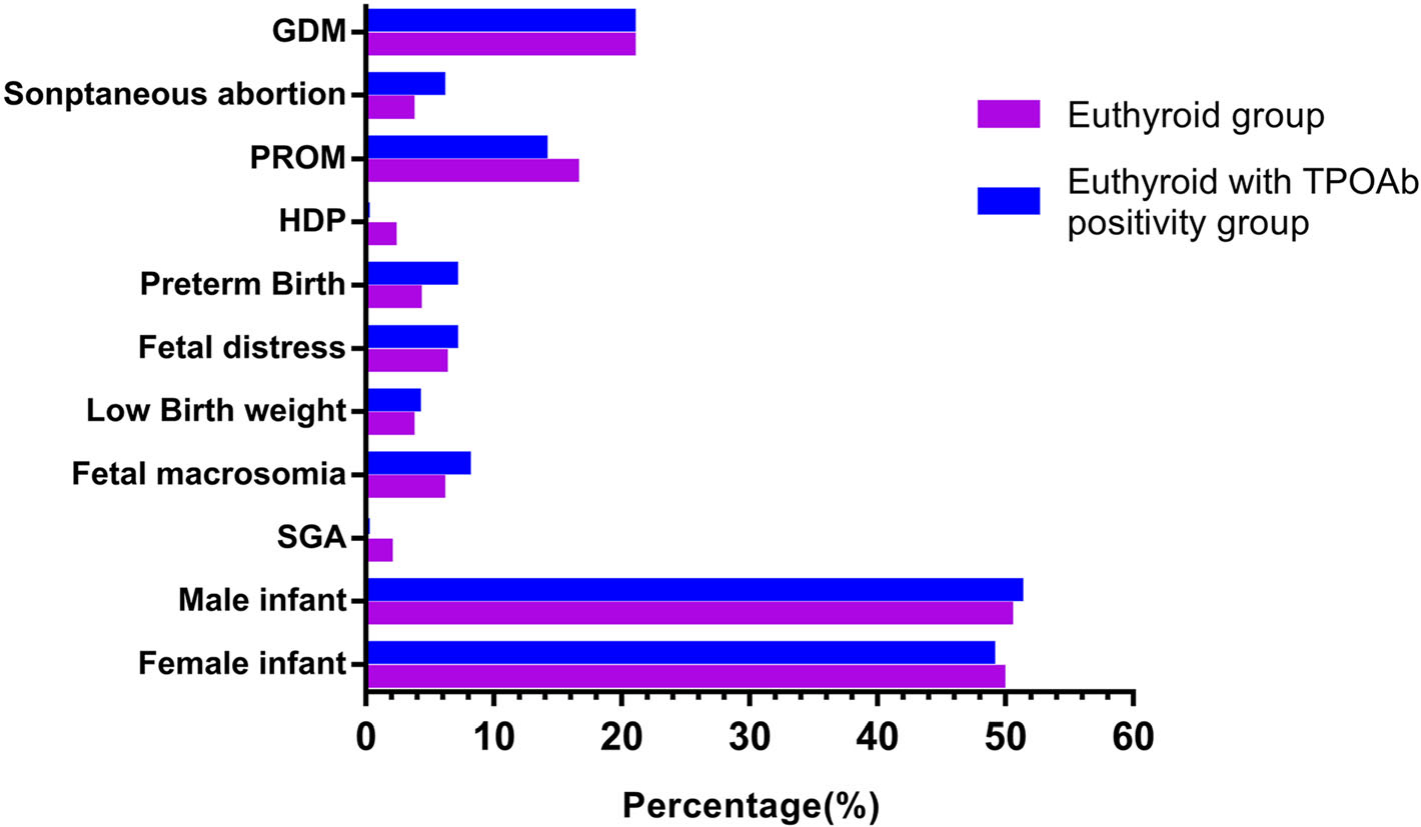
Subject’s pregnancy and fetal outcomes in the Euthyroid group and the Euthyroid women with TPOAb positivity group. GDM, gestational diabetes; PROM, premature rupture of membranes; HDP, hypertensive disorders of pregnancy; SGA, small for gestational age

The pregnancy-related and fetal outcomes in the two groups are summarized in Table 2 and Fig. 2. The incidences of spontaneous abortion and preterm births were higher in the TPOAb-positive euthyroid women than in the euthyroid group; however, the difference was not significant (5.9% vs 3.5%, *P* = 0.215; 6.9% vs 4.1%. *P* = 0.183, respectively). In this study, four iatrogenic preterm birth were excluded (2 cases of placenta previa, 1 case of fetal brain edema, and 1 case of severe eclampsia). In the euthyroid group, the incidence of HDP and SGA were 2.1 and 1.8% respectively. In the TPOAb-positive euthyroid group, there were no pregnant women with HDP and no SGA infants. No significant differences were found in the incidence of GDM, PROM, fetal distress, low birth weight, fetal macrosomia, female/male infant ratio, birthweight, or gestational age at birth between the two groups.

**Table 2:** Subject’s pregnancy and fetal outcomes in Euthyroid group and Euthyroid women with TPOAb positivity group

	Euthyroid group (*n* = 837)	Euthyroid women with TPOAb positivity group (*n* = 101)	Statistics	P
GDM (%)	174 (20.8%)	21 (20.8%)	0.000	0.999
Spontaneous abortion (%)	29 (3.5%)	6 (5.9%)	1.538	0.215
PROM (%)	137 (16.4%)	14 (13.9%)	0.419	0.517
HDP(%)	18 (2.1%)	NA		
Preterm Birth (%)	34 (4.1%)	7 (6.9%)	1.774	0.183
Fetal distress (%)	51 (6.1%)	7 (6.9%)	0.109	0.741
Low Birth weight (%)	29 (3.5%)	4 (4.0%)	0.063	0.802
Fetal macrosomia (%)	49 (5.9%)	8 (7.9%)	0.674	0.412
SGA (%)	15 (1.8%)	NA		
Infant				
Female infant (%)	399 (49.7%)	46 (48.9%)	0.019	0.890
Male infant (%)	404 (50.3%)	48 (51.1%)		
Birthweight (Kg)	3.35 (3.08,3.61)	3.32 (3.02,3.60)	−0.856	0.392
Gestational age at birth (weeks)	39 (38,40)	39 (38,40)	−0.760	0.447

**Statistics:** Birthweight and gestational age at birth for Mann-Whitney U test; pregnancy and fetal outcomes for chi-square test or Fisher test

Continuous data are expressed as median (interquartile range). Categorical data are expressed as numbers (percentages) of cases

*GDM* gestational diabetes, *PROM* premature rupture of membranes, *HDP* hypertensive disorders of pregnancy, *SGA* small for gestational age

### Univariable logistic regression analysis

Results from the univariable logistic regression analysis performed with demographic parameters as independent variables and poor pregnancy and fetal outcomes as categorical dependent variables showed that GDM was associated with maternal age ≥ 35 years (OR: 2.055, 95% CI: 1.418–2.978) and BMI ≥ 24 kg/m^2^ (OR: 2.284, 95% CI: 1.568–3.327). Fetal macrosomia was associated with BMI ≥ 24 kg/m^2^ (OR: 2.543, 95% CI: 1.359–4.761). Incidences of fetal distress were associated with multipara (OR: 0.323, 95% CI: 0.161–0.649). There was no correlation between spontaneous abortion, PROM, HDP, preterm birth, low birth weight, SGA infant and the demographic parameters (Table 3).

**Table 3:** Univariable logistic regression analysis with demographic parameters as independent variables and pregnancy and fetal outcomes as categorical dependent variables

pregnancy and fetal outcomes	Age ≥ 35 years		BMI ≥ 24 kg/m^2^		Multipara		History of spontaneous abortion	
Independent variables	OR (95% CI)	p	OR (95% CI)	p	OR (95% CI)	p	OR (95% CI)	p
GDM	2.055 (1.418–2.978)	0.000*	2.284 (1.568–3.327)	0.000*	1.317 (0.948–1.830)	0.101	0.922 (0.571–1.490)	0.740
Spontaneous abortion	1.937 (0.934–4.014)	0.076	0.853 (0.320–2.276)	0.751	1.108 (0.540–2.273)	0.785	1.604 (0.687–3.744)	0.275
PROM	0.648 (0.396–1.061)	0.085	0.816 (0.512–1.303)	0.395	0.737 (0.507–1.072)	0.737	0.568 (0.311–1.037)	0.066
HDP	1.661 (0.584–4.720)	0.341	2.572 (0.982–6.733)	0.054	0.803 (0.299–2.160)	0.664	1.877 (0.611–5.826)	0.270
Preterm Birth	0.874 (0.381–2.005)	0.751	1.236 (0.574–2.661)	0.588	1.128 (0.587–2.167)	0.718	0.889 (0.346–2.337)	0.828
Fetal distress	0.756 (0.365–1.568)	0.453	0.912 (0.449–1.853)	0.799	0.323 (0.161–0.649)	0.001*	1.105 (0.891–3.362)	0.105
Low Birth weight	0.947 (0.385–2.329)	0.906	1.108 (0.471–2.606)	0.813	0.621 (0.284–1.359)	0.233	0.894 (0.309–2.587)	0.836
Fetal macrosomia	0.906 (0.512–2.126)	0.906	2.543 (1.359–4.761)	0.004*	1.060 (0.585–1.922)	0.848	1.637 (0.798–3.359)	0.179
SGA	1.095 (0.305,3.923)	0.890	1.598 (0.495,5.158)	0.433	0.243 (0.054,1.082)	0.063	1.468 (0.468,6.06)	0.425

*P < 0.05

*GDM* gestational diabetes, *PROM* premature rupture of membranes, *HDP* hypertensive disorders of pregnancy, *SGA* small for gestational age

### Multivariate logistic regression analysis

Logistic regression analysis with poor pregnancy and fetal outcomes as the categorical dependent variables showed that euthyroid women with TPOAb positivity did not have a higher risk of poor pregnancy or fetal outcomes, including GDM, spontaneous abortion, PROM, preterm birth, fetal distress, low birth weight, and fetal macrosomia. However, in euthyroid women with a female fetus, TPOAb positivity was independently associated with preterm birth (OR: 4.511, 95% CI: 1.075–18.926) after adjustment for demographic parameters, HbA1c, and TSH. No significant relationship was found between TPOAb positivity and preterm birth among euthyroid women with a male fetus (Table 4).

**Table 4:** Logistic regression analysis with euthyroid women with TPOAb positivity as independent variables and poor pregnancy and fetal outcomes as categorical dependent variables

GDM	Total		Pregnant women with a female fetus		Pregnant women with a male fetus	
	OR (95% CI)	P	OR (95% CI)	P	OR (95% CI)	p
Model 1	0.991 (0.558–1.760)	0.975	0.596 (0.239–1.483)	0.239	1.696 (0.801–3.591)	0.168
Model 2	1.282 (0.610–2.694)	0.512	0.690 (0.215–2.218)	0.534	2.349 (0.831–6.638)	0.107
Spontaneous abortion						
Model 1	1.925 (0.709–5.222)	0.199				
Model 2	1.083 (0.227–5.171)	0.920				
PROM						
Model 1	0.757 (0.399–1.436)	0.383	0.641 (0.239–1.717)	0.197	0.914 (0.386–2.168)	0.914
Model 2	0.476 (0.181–1.250)	0.132	0.835 (0.264–2.640)	0.758	0.391 (0.084–1.831)	0.233
Preterm Birth						
Model 1	1.765 (0.713–4.370)	0.219	3.650 (1.195–11.154)	0.023*	1.487 (0.415–5.328)	0.543
Model 2	2.202 (0.699–6.937)	0.178	4.511 (1.075–18.926)	0.04*	0.856 (0.103–7.116)	0.886
Fetal distress						
Model 1	1.680 (0.755–3.736)	0.204	2.669 (0.999–7.129)	0.05	0.826 (0.183–3.734)	0.804
Model 2	1.084 (0.307–3.820)	0.900	1.902 (0.371–9.472)	0.440	0.769 (0.091–6.487)	0.809
Low Birth weight						
Model 1	1.233 (0.480–3.171)	0.663	1.117 (0.246–5.074)	0.886	1.887 (0.394–9.023)	0.427
Model 2	1.128 (0.251–5.073)	0.875	0.617 (0.072–5.314)	0.661	2.467 (0.486–12.532)	0.276
Fetal macrosomia						
Model 1	0.413 (0.098,1.745)	0.229	0.54 (0.069–4.218)	0.557	0.385 (0.050–2.944)	0.358
Model 2	0.401 (0.094–1.707)	0.216	0.504 (0.63–4.030)	0.518	0.375 (0.049–2.893)	0.347

*P < 0.05

Model 1: Adjusted for age, BMI, parity, and history of spontaneous abortion

Model 2: Additionally, adjusted for HbA1c and TSH

*BMI* body-mass index, *TSH* thyroid-stimulating hormone, *HbA1c* glycated hemoglobin, *GDM* gestational diabetes, *PROM* premature rupture of membranes

## Discussion

This study aimed to investigate the association between TPOAb positivity and pregnancy-related and fetal outcomes in euthyroid women. The primary finding is that TPOAb positivity was not associated with an increased risk of poor pregnancy or fetal outcomes, including preterm birth, GDM, spontaneous abortion, PROM, fetal distress, low birth weight, and fetal macrosomia in euthyroid women. However, in euthyroid pregnant women with a female fetus, TPOAb positivity was independently associated with preterm birth after adjustment for demographic parameters, HbA1c, and TSH.

TPOAb positivity is present in 6 to 8.8% of pregnant women [7, 18], and TAI and SCH during pregnancy are associated with poor pregnancy and fetal outcomes [19, 20]. The upper normal cutoff limit for TSH was set at 4.0 mU/L during pregnancy instead of 2.5 mU/L as per the 2017 ATA guidelines [3]. However, LT4 replacement therapy may be considered for TPOAb-positive women with TSH > 2.5 mU/L and below the upper limit of the pregnancy-specific reference range [3]. The association between TPOAb positivity and poor pregnancy and fetal outcomes in euthyroid women remains controversial because evidence from several prospective cohort studies that investigated outcomes such as spontaneous abortions, GDM, and preterm births and other rarer outcomes such as PROM, fetal distress, low birth weight, fetal macrosomia, and SGA infant, were inconclusive [7, 21]. Since it may play a role in maternal physiological processes during pregnancy, the secondary objective in the present study was to compare the effect of TPOAb positivity on pregnancy and fetal outcomes according to the sex of the fetus. The results indicate that TPOAb positivity was independently associated with preterm birth in euthyroid pregnant women with a female fetus.

Preterm birth complicates 5 to 15% of births worldwide and is the leading cause of morbidity and mortality in children younger than 5 years [7, 22]. Thus, it is very important to identify its risk factors. Hypothyroidism and SCH were found to be associated with preterm births in previous studies [7, 23, 24]. However, it remains unclear whether TPOAb positivity is a risk factor for preterm birth in euthyroid women. In this study, TPOAb positivity was not associated with a higher risk of preterm birth except when the sex of the fetus was considered; in particular, TPOAb positivity in women with a female fetus was associated with preterm birth. Therefore, the risk of preterm birth in euthyroid women with TPOAb positivity should be clinically emphasized since consistent results have been obtained in previous studies. In a birth cohort study in Ma’anshan, which is an iodine-sufficient area of China, TPOAb positivity was associated with a higher risk of preterm birth [25]. A meta-analysis showed pregnant women with TPOAb positivity had a substantial risk of preterm delivery compared with the reference group (RR: 1.69, 95% CI 1.19–2.41, *P* = 0.003); however, this relationship was not found in pregnant women with TGAb positivity [26]. A separate meta-analysis found that TPOAb-positive women had a higher risk of preterm birth than TPOAb-negative women (6.6% vs 4.9%, respectively; OR, 1.33 [95% CI, 1.15–1.56]) [7]. The two meta-analyses, which included cohort studies, suggest that TPOAb positivity is associated with preterm birth. Although the subgroup analysis of the fetus sex in this study found that TPOAb positivity was only associated with preterm birth in pregnant women with a female fetus, TPOAb-positive euthyroid women should be emphasized in clinical practice. Moreover, the potential benefit of LT4 therapy remains unclear. A meta-analysis [27] found that LT4 supplementation reduced the risks of pregnancy loss and preterm birth in women with TAI. However, a separate meta-analysis [28] showed that there was no definitive evidence that LT4 supplementation improved the pregnancy outcomes in euthyroid women with TAI.

On the contrary, published data have suggested that TAI has no association with preterm births. Among women with 1–2 previous pregnancy losses, TAI was not associated with an increased risk of preterm delivery in euthyroid women [29]. Chen et al. showed that there was no difference in the incidence of preterm births between TPOAb-positive with or without TGAb positivity and euthyroid women [21]. A secondary analysis of a prospective cohort in Denmark reported that there was more than double the number of pregnant women with thyroid antibody positivity as part of an iodine fortification program without an increase in preterm birth-rate [30]. The disparate results may be due to multiple factors, including the year the research was conducted, the iodine-related nutrition status of the study participants, the definitions and measures for the outcomes, and the demographic situation of the enrolled pregnant women. In addition, none of the studies conducted subgroup analysis by sex of the fetus. Further randomized controlled trials and fundamental studies are warranted to confirm the pregnancy outcomes and treatment of euthyroid women with TPOAb positivity.

The pathophysiological mechanism of preterm birth caused by TAI remains unclear. Some preterm births are caused by impaired thyroidal response to HCG, inflammation, or accompanied by other autoimmune diseases and obstetric complications. TPOAb positivity is an indication of an impaired thyroidal response to HCG and an inadequate FT4 response to HCG has been associated with a higher risk of preterm birth [31]. Interleukin-6 (IL-6) levels are significantly higher in pregnant women with thyroid antibody positivity, suggesting that TAI is associated with low-grade inflammation [32]. A few studies showed a significant relationship between increased IL-6 levels and the risk of preterm birth [33]. Furthermore, many pregnant women with TAI often suffer from other autoimmune diseases, such as systemic lupus erythematosus and antiphospholipid syndrome, which are well-known causes of preterm births. Several studies found that TAI was associated with the risk of placental abruption, preeclampsia, and intrauterine growth restriction, obstetric complications that are known to be involved in preterm births [15, 31].

In this study, TPOAb positivity was independently associated with preterm birth only in pregnant women with a female fetus. Some studies have suggested differences in pregnancy and fetal outcomes according to the sex of the fetus. For example, fetal sex exerts a differential effect on the placental pathology that mediates severe preeclampsia and/or intrauterine growth restriction [34]. A recent meta-analysis found preterm preeclampsia (delivered < 34 weeks) was even more prevalent among pregnancies with a female fetus compared with pregnancies with a male fetus [35]. Mitchell et al. [36] suggested that examination of the moderating role of fetal sex differences among women with adverse pregnancy outcomes (e.g., preterm birth) in future studies would be informative. However, the mechanism associating preterm birth caused by TAI in pregnant women with a female fetus is still not clear. Lee et al. showed that TSH levels were significantly higher in females than in males (75 and 41%, respectively; *p* = 0.037) and that the genetic influences on individual TSH levels were more prominent in females than in males [5]. It is known that females are more likely than males to develop thyroid disease. It has been shown that women carrying a female fetus had a greater inflammatory response after an immune challenge than those carrying a male fetus [36]. Pregnancies with a female fetus have a greater predisposition to preterm birth associated with hypertension [35, 37] and TAI may be involved in this process [38]. In addition, some studies have shown that male fetuses may be more vulnerable to certain poor outcomes. For example, in a retrospective study that included pregnant women with preeclampsia, among the preterm infants born, male fetuses were more likely to be impaired in growth than female fetuses [39]. In another study, male fetuses may have been more vulnerable to intrauterine adversity than female fetuses [40]. However, this study mainly indicated that males who were small for gestational age at birth were more likely to have decreased physical development after birth. Therefore, the association of fetal sex on pregnancy-related and fetal outcomes should arouse the concern of researchers.

Previous studies regarding TPOAb positivity and pregnancy-related and fetal outcomes have yielded mixed results. In this study, there was no significant difference in the incidence of GDM, spontaneous abortion, PROM, fetal distress, low birth weight, or fetal macrosomia between the two groups. Our results are consistent with the results of several studies [21, 41, 42]. However, some studies have found an association between TAI and other poor pregnancy and fetal outcomes. For example, one meta-study showed that there was an association between thyroid antibodies and the risk of GDM [43]. Chen et al. showed that TPOAb positivity was associated with PROM and low birth weight [21]. The different findings between studies may be due to the varying methodology and study populations. Therefore, inconsistent evidence remains and the relationship between pregnancy-related and fetal outcomes and TPOAb positivity in euthyroid women needs to be studied further.

In this study, univariable logistic regression analysis showed that GDM was associated with advanced maternal age and high maternal BMI, fetal macrosomia was associated with high maternal BMI, and fetal distress was associated with first live birth. Therefore, the poor pregnancy-related and fetal outcomes were associated with demographic parameters and not thyroid disorders or TAI. It should be noted that there are multiple contributing factors between poor pregnancy-related and fetal outcomes. It may be because the impact of TPOAb positivity on poor pregnancy and fetal outcomes in euthyroid women varies with the sex of the fetus, and demographic and obstetric parameters, according to different studies [21, 26, 42].

Several strengths of this study need to be underscored. First, this was a prospective cohort study to investigate the relationship between TPOAb positivity and pregnancy-related and fetal outcomes in euthyroid women. Secondly, numerous outcome variables were included such as spontaneous abortion, GDM, HDP and preterm birth, as well as other less frequently studied outcomes, such as PROM, fetal distress, low birth weight, fetal macrosomia, and SGA infant. Thirdly, we performed a subgroup analysis based on fetus sex and found that there was a difference according to sex on the occurrence of preterm birth. Fetus sex examinations are strictly banned unless medically necessary in China; therefore, the risks of preterm birth in all euthyroid women with TPOAb positivity should be emphasized in clinical practice. This study also had some limitations to consider. Firstly, this study was a single-center cohort study which limited the generalizability of the results and involved only a few demographic parameters that may influence pregnancy-related and fetal outcomes. Other demographic parameters, such as socioeconomic status, education level, and nutritional status, were not considered, although data on these factors would help to guide services for pregnant women in this region. Secondly, women in this study were enrolled at 4 to 8 weeks’ gestation; hence, spontaneous abortions that occurred early in the first trimester of pregnancy might have been missed. However, in Beijing, it is common for women to seek prenatal care during the first 4 weeks of gestation. Thus, only a very small number of patients who underwent spontaneous abortions might have been missed. In addition, some subgroup analyses involved a small study population; hence, the lack of association of TPOAb status and preterm birth and miscarriages may be due to type 2 statistical error. Finally, no iodine intake measurements were taken for the study; however, there is universal salt iodination in China and the city of Beijing is an iodine-sufficient region [44].

## Conclusions

TPOAb positivity was not associated with poor pregnancy-related or fetal outcomes in euthyroid women. However, in euthyroid women with a female fetus, TPOAb positivity was strongly associated with preterm birth. The risk of preterm birth in the euthyroid women with TPOAb positivity should be emphasized in clinical practice.

## Data Availability

The datasets used and/or analyzed during the current study are available from the corresponding author on reasonable request.

## Abbreviations

BMI: Body mass index
FT4: Free thyroxine
GDM: Gestational diabetes
GS: Blood glucose
HCG: Human chorionic gonadotropin
HbA1c: Glycated hemoglobin
Hcy: Homocysteine
HDP: Hypertensive disorders of pregnancy
LDL: Low-density lipoprotein cholesterol
PROM: Precmature rupture of membranes
SCH: Subclinical hypothyroidism
SGA: Small for gestational age
TAI: Thyroid autoimmunity
TGAb: Thyroglobulin antibody
TPOAb: Anti-thyroid peroxidase antibody
TSH: Thyroid-stimulating hormone
UA: Uric acid
CV: Coefficient of variability
IL-6: Interleukin-6
IQR: Interquartile range
LT4: Levothyroxine
